# Information of Growth Traits Is Helpful for Genetic Evaluation of Litter Size in Pigs

**DOI:** 10.3390/ani14182669

**Published:** 2024-09-13

**Authors:** Hui Yang, Lei Yang, Jinhua Qian, Lei Xu, Li Lin, Guosheng Su

**Affiliations:** 1 Department of Animal Science, Fujian Vocational College of Agriculture, Fuzhou 350007, China; xulei0328@outlook.com (L.X.); ll-ny@outlook.com (L.L.); 2 Suzhou Aspire Agritech Consulting Co., Ltd., Suzhou 215000, China; lyang@aspire-ag.com; 3 College of Animal Science and Technology, Yangzhou University, Yangzhou 225009, China; lqian@aspire-ag.com; 4 Center for Quantitative Genetics, Aarhus University, 8000 Aarhus, Denmark

**Keywords:** genetic correlation, genetic evaluation, heritability, litter size, pig, multitrait model

## Abstract

**Simple Summary:**

Litter size is an important trait in pig production. Selection accuracy for litter size is expected to be increased by genetic evaluation using data of production traits as additional information. This study investigated the improvement of genetic evaluation for litter size using a multitrait model including production traits. The multitrait model used in this study allows us to account for environmental correlation between litter size and production traits in the situation that one individual has only one record for a production trait while multiple records for litter size. The results show that the multitrait model including growth trait can improve genetic evaluation for litter size considerably. Therefore, it is recommended to use the multitrait model with data of both reproduction and production traits for routine genetic evaluation in pig breeding programs.

**Abstract:**

Litter size is an important trait in pig production. But selection accuracy for this trait is relatively low, compared with production traits. This study, for the first time, investigated the improvement of genetic evaluation of reproduction traits such as litter size in pigs using data of production traits as an additional information source. The data of number of piglets born alive per litter (NBA), age at 100 kg of body weight (Age100), and lean meet percentage (LMP) in a Yorkshire population were analyzed, using either a single-trait model or the multitrait model that allows us to account for environmental correlation between reproduction and production traits in the situation that one individual has only one record for a production trait while multiple records for a reproduction trait. Accuracy of genetic evaluation using single-trait and multitrait models were assessed by model-based accuracy (R_m_) and validation accuracy (R_v_). Two validation scenarios were considered. One scenario (Valid_r1) was that the individuals did not have a record of NBA, but Age100 and LMP. The other (Valid_r2) was that the individuals did not have a record for all the three traits. The estimate of heritability was 0.279 for Age100, 0.371 for LMP, and 0.076 for NBA. Genetic correlation was 0.308 between Age100 and LMP, 0.369 between Age100 and NBA, and 0.022 between LMP and NBA. Compared with the single-trait model, the multitrait model including Age100 increased prediction accuracy for NBA by 3.6 percentage points in R_m_ and 5.9 percentage points in R_v_ for the scenario of Valid_r1. The increase was 1.8 percentage points in R_m_ and 3.8 percentage points in R_v_ for the scenario of Valid_r2. Age100 also gained in the multitrait model but was smaller than NBA. However, LMP did not benefit from a multitrait model and did not have a positive contribution to genetic evaluation for NBA. In addition, the multitrait model, in general, slightly reduced level bias but not dispersion bias of genetic evaluation. According to these results, it is recommended to predict breeding values using a multitrait model including growth and reproduction traits.

## 1. Introduction

Litter size is an important trait in pig production. However, selection accuracy for this trait is relatively low compared with production traits mainly due to two reasons. The first is the low heritability of litter size. Previous studies have shown that heritability estimates of litter size are around 0.10 [1,2,3,4]. The second is that breeding animals are usually selected after growth performance test but before obtaining records of litter size.

Multitrait models have been widely used for genetic evaluation in animal breeding programs. The advantage of a multitrait model is that it allows the use of information from genetic correlated traits to predict breeding value of the trait of interest. Multitrait models greatly benefit a trait that has low heritability and/or small number of phenotypic records while its genetic correlated traits have high heritability and/or large number of phenotypic records. Previous studies have reported an unfavorable genetic correlation between growth and reproduction traits in pig populations [2,5,6,7], and heritability of production traits are moderately high [1,2,8,9]. Moreover, the number of pigs with production records is usually much larger than the number of pigs with reproduction records. Furthermore, in general, at the time of final selection, pigs already have production records but not reproduction records. Therefore, it is hypothesized that genetic evaluation for reproduction traits can be improved using a multitrait model including production traits. However, in pig breeding programs, genetic evaluation of production traits and reproductive traits are usually performed separately. To our knowledge, there are no studies in the literature that investigate the improvement of genetic evaluation of reproductive traits by incorporating production trait information in pig populations.

The objective of this study is to estimate genetic parameters of production traits and reproduction traits, and assess the benefit of genetic evaluation for reproduction trait using production traits as additional information. The analysis was performed using a novel multitrait model allowing us to handle single records for a production trait while multirecords for a reproduction trait, based on the data from a Yorkshire population.

## 2. Materials and Methods

### 2.1. Data

The traits in the analysis were number of piglets born alive per litter (NBA), age at 100 kg of body weight (Age100), and lean meet percentage (LMP) in a Yorkshire population. LMP was estimated from ultrasonic measurements of backfat thickness and loin muscle area. There were 35,875 pigs with records of Age100 and LMP collected from breeding herds. The NBA included 15,636 records from 6049 sows, collected from both breeding herds and some multiplier herds. The breeding herds were selected for an index including NBA, Age100, and LMP100. Sow management and NBA recording are the same in both breeding herd and multiplier herd. In both breeding and multiplier herds, each sow is artificially inseminated twice with the same boar. During gestation, the sows fed twice daily. The daily feed allowances of the sows were based on their body condition. NBA was calculated immediately after farrowing. Pedigree was traced back to five generations, according to the study by Yang and Su [10]. In total, there were 45,924 individuals in the pedigree data.

### 2.2. Statistical Models

The main objective of this study was to investigate the improvement of genetic evaluation for NBA by using growth trait data as additional information source, compared with using NBA information alone. Therefore, we estimated variance components and breeding values using three animal models. A single-trait model uses information only from the trait itself. A two-trait model estimates breeding value for a trait using information not only from the trait itself, but also the information of another trait in the model. A three-trait model estimates breeding value for a trait using information of all the three traits in this study. Unlike a sire model which fits a unique breeding value for each sire, an animal model fits a unique breeding value for each animal.

(1) Single-trait model

The single-trait model for Age100 and LMP includes effect of herd-year-month of birth, sex effect, individual additive genetic effect, litter effect, and residual effect. In matrix-vector form, it is

(1) $$y=Xb+Z_{a}a+Z_{l}l+e$$

where **y** is the vector of observations for Age100 or LMP, **b** is the vector of fixed effects which include herd-year-month of birth and sex, **a** is the vector of additive genetic effects, **l** is the vector of litter effects, **e** is the vector of residuals, and **X**, **Z_a_**, and **Z_l_** are the coefficient matrixes linking **b**, **a**, and **l** to **y**.

The single-trait model for NBA includes effects of herd-year-month of farrowing, parity, litter type, age of sow at first farrowing and farrowing interval, sow additive genetic effect, sow permanent environmental effect and residual effect. In matrix-vector form, it is

(2) $$y=Xb+Z_{a}a+Z_{pe}pe+e$$

where **y** is the vector of observations for NBA; **b** is the vector of fixed effects, which include herd-year-season of farrowing, parity, litter type (pure or crossed litter), and regressions on first and second order of age of sow at first farrowing, and first and second order of interval between the current farrowing and the previous farrowing; **a** is the vector of additive genetic effects; **pe** is the vector of permanent environmental effects of sows; **e** is the vector of residuals; and **X**, **Z_a_**, and **Z_pe_** are the coefficient matrixes linking **b**, **a**, and **pe** to **y**.

In the above models, it is assumed that the random effects have the following normal distributions:

$$a\sim N(0,{A\sigma }_{a}^{2}),\;l\sim N(0,{I\sigma }_{l}^{2}),\;pe\sim N(0,{I\sigma }_{pe}^{2}),\;\mathrm{and}\;e\sim N(0,{I\sigma }_{e}^{2})$$

where $\sigma_{a}^{2}$, $\sigma_{l}^{2}$, $\sigma_{pe}^{2}$, and $\sigma_{e}^{2}$ are variances of additive genetic effect, litter effect, permanent effect, and residual effect, respectively, **A** is the genetic relationship matrix calculated based on pedigree, and **I** is an identity matrix.

(2) Two-trait model

The two-trait model for Age100 and LMP (subscripted as 1 and 2 below) has the same form as the corresponding single-trait model.

(3) $$\left[\begin{array}{c} y_{1}\\ y_{2} \end{array}\right]=\left[\begin{array}{c} X_{1}\;\;\;\;\;0\\ 0\;\;\;\;\;X_{2} \end{array}\right]\left[\begin{array}{c} b_{1}\\ b_{2} \end{array}\right]+\left[\begin{array}{c} Z_{a1}\;\;\;\;0\\ 0\;\;\;\;Z_{a2} \end{array}\right]\left[\begin{array}{c} a_{1}\\ a_{2} \end{array}\right]+\left[\begin{array}{c} Z_{l1}\;\;\;\;0\\ 0\;\;\;\;Z_{l2} \end{array}\right]\left[\begin{array}{c} l_{1}\\ l_{2} \end{array}\right]+\left[\begin{array}{c} e_{1}\\ e_{2} \end{array}\right]$$

It is assumed that the random effects of the two-trait model have the following normal distributions:

$$\begin{array}{l} \left(\begin{array}{c} a_{1}\\ a_{2} \end{array}\right)\sim N\left(0,\left[\begin{array}{c} \sigma_{a1}^{2}\;\;\sigma_{a12}\\ \sigma_{a12}\;\sigma_{a2}^{2}\; \end{array}\right]⊗A\right),\;\left(\begin{array}{c} l_{1}\\ l_{2} \end{array}\right)\sim N\left(0,\left[\begin{array}{c} \sigma_{l1}^{2}\;\;\sigma_{l12}\\ \sigma_{l12}\;\sigma_{l2}^{2}\; \end{array}\right]⊗I\right),\\ \mathrm{and}\;\left(\begin{array}{c} e_{1}\\ e_{2} \end{array}\right)\sim N\left(0,\left[\begin{array}{c} \sigma_{e1}^{2}\;\;\sigma_{e12}\\ \sigma_{e12}\;\sigma_{e2}^{2}\; \end{array}\right]⊗I\right) \end{array}$$

where $\sigma_{a12},\sigma_{l12},\;and\;\sigma_{e12}$ are covariance between the two traits for additive genetic, litter, and residual effects, respectively.

The two-trait model for NBA and a production trait (Age100 or LMP) cannot be constructed by simply combining the two single-trait models of the two traits, because there is only one record for a production trait but multiple records for NBA in an individual. For an individual with both NBA and production trait records, the individual permanent environment affects both NBA and production performance. Thus, there exists a permanent environment covariance between NBA and production traits. On other hand, NBA and production traits are measured at different times; their residual effects are assumed independent from each other. The model for NBA can separate permanent environment effect and residual effect because an individual (sow) has multiple records (each for one parity), whereas the typical production trait model cannot because there is only one record for one production trait in one individual. In other words, the residual effect in a typical animal model for a production trait is, in fact, a mixture of individual permanent effect and residual effect. This makes it difficult to construct the covariance of environment effects between NBA and a production trait. A solution for it is to forcibly divide the residual effect of a production trait in model (1) into a permanent environment effect and a residual effect, i.e., $e=pe+e^{*}$. This make it possible to model the covariance of environment effects between NBA and a production trait by fitting a covariance between *pe* of NBA and *pe* of a production trait. Thus, letting y_1_ denote Age100 or LMP and y_2_ denote NBA, the two-trait model is constructed in the following form.

(4) $$\left[\begin{array}{c} y_{1}\\ y_{2} \end{array}\right]=\left[\begin{array}{c} X_{1}\;\;\;\;\;0\\ 0\;\;\;\;\;X_{2} \end{array}\right]\left[\begin{array}{c} b_{1}\\ b_{2} \end{array}\right]+\left[\begin{array}{c} Z_{a1}\;\;\;\;0\\ 0\;\;\;\;Z_{a2} \end{array}\right]\left[\begin{array}{c} a_{1}\\ a_{2} \end{array}\right]+\left[\begin{array}{c} Z_{l1}\;\;\;\;0\\ 0\;\;\;\;\;\;\;0 \end{array}\right]\left[\begin{array}{c} l_{1}\\ 0 \end{array}\right]+\left[\begin{array}{c} Z_{pe1}\;\;\;\;0\\ 0\;\;\;\;Z_{pe2} \end{array}\right]\left[\begin{array}{c} {pe}_{1}\\ {pe}_{2} \end{array}\right]+\left[\begin{array}{c} e_{1}^{*}\\ e_{2} \end{array}\right]$$

In such a model, the permanent effect and the remaining residual effect for the production trait are not identifiable. Therefore, the variance of e_1_^*^ is fixed to be about 1% of phenotypic variance. The 1% is arbitrary, but the sum of $\sigma_{pe1}^{2}$ and $\sigma_{{e1}^{*}}^{2}$ is equivalent to $\sigma_{e1}^{2}$. However, the value should be small enough to ensure the whole environmental covariance between the production trait and NBA being accounted for by the **pe** term. It is assumed that

$$\begin{array}{l} \left(\begin{array}{c} a_{1}\\ a_{2} \end{array}\right)\sim N\left(0,\left[\begin{array}{c} \sigma_{a1}^{2}\;\;\sigma_{a12}\\ \sigma_{a12}\;\sigma_{a2}^{2}\; \end{array}\right]⊗A\right),\;l\sim N(0,{I\sigma }_{l}^{2}),\;\left(\begin{array}{c} {pe}_{1}\\ {pe}_{2} \end{array}\right)\sim N\left(0,\left[\begin{array}{c} \sigma_{pe1}^{2}\;\;\sigma_{pe12}\\ \sigma_{pe12}\;\sigma_{pe2}^{2}\; \end{array}\right]⊗I\right),\\ \mathrm{and}\;\left(\begin{array}{c} e_{1}^{*}\\ e_{2} \end{array}\right)\sim N\left(0,\left[\begin{array}{cc} \sigma_{{e1}^{*}}^{2} & 0\\ 0 & \sigma_{e2}^{2} \end{array}\right]⊗I\right) \end{array}$$

(3) Three-trait model

The three-trait model is the expansion of the two-trait model including NBA. Letting y_1_, y_2_, and y_3_ denote Age100, LMP, and NBA, the three-trait model is

(5) $$\left[\begin{array}{c} y_{1}\\ y_{2}\\ y_{3} \end{array}\right]=\left[\begin{array}{c} X_{1}\;0\;\;0\\ 0\;\;X_{2}\;0\\ 0\;\;0\;X_{3} \end{array}\right]\left[\begin{array}{c} b_{1}\\ b_{2}\\ b_{3} \end{array}\right]+\left[\begin{array}{c} Z_{a1}\;\;0\;\;\;0\\ 0\;\;\;Z_{a2}\;\;0\\ 0\;\;\;0\;\;Z_{a3} \end{array}\right]\left[\begin{array}{c} a_{1}\\ a_{2}\\ a_{3} \end{array}\right]+\left[\begin{array}{c} Z_{l1}\;0\;\;\;0\\ 0\;\;\;Z_{l2}\;0\\ 0\;\;\;0\;\;\;\;0 \end{array}\right]\left[\begin{array}{c} l_{1}\\ l_{2}\\ 0 \end{array}\right]+\left[\begin{array}{c} Z_{pe1}\;0\;\;\;\;0\\ 0\;\;Z_{pe2}\;\;0\\ 0\;\;\;0\;\;Z_{pe3} \end{array}\right]\left[\begin{array}{c} {pe}_{1}\\ {pe}_{2}\\ {pe}_{3} \end{array}\right]+\left[\begin{array}{c} e_{1}^{*}\\ e_{2}^{*}\\ e_{2} \end{array}\right]$$

The assumption of distributions of random effects has the same form as the two-trait model including NBA.

Heritability for each trait was estimated based on the variance components from the single-trait models. Heritability for Age100 and LMP was calculated as

(6) $$h^{2}=\frac{\sigma_{a}^{2}}{\sigma_{p}^{2}}=\frac{\sigma_{a}^{2}}{\sigma_{a}^{2}+\sigma_{l}^{2}+\sigma_{e}^{2}}$$

and heritability for NBA was calculated as

(7) $$h^{2}=\frac{\sigma_{a}^{2}}{\sigma_{p}^{2}}=\frac{\sigma_{a}^{2}}{\sigma_{a}^{2}+\sigma_{pe}^{2}+\sigma_{e}^{2}}$$

Genetic correlation (r_a_) and phenotypic correlation (r_p_) between each pair of the traits were estimated based on (co)variances from the three-trait model:

(8) $$r_{a}=\frac{\sigma_{aij}}{\sqrt{\sigma_{ai}^{2}\sigma_{aj}^{2}}},\;r_{p}=\frac{\sigma_{pij}}{\sqrt{\sigma_{pi}^{2}\sigma_{pj}^{2}}}$$

where phenotypic covariance σ_pij_ between Age100 and LMP was defined as $\sigma_{pij}=\sigma_{aij}+\sigma_{lij}+\sigma_{peij}$, phenotypic covariance σ_pij_ between NBA and Age100 or LMP was defined as $\sigma_{pij}=\sigma_{aij}+\sigma_{peij}$, phenotypic variance of Age100 and LMP was $\sigma_{pi}^{2}=\sigma_{ai}^{2}+\sigma_{li}^{2}+\sigma_{pei}^{2}+\sigma_{ei*}^{2}$, and phenotypic variance of NBA was $\sigma_{pi}^{2}=\sigma_{ai}^{2}+\sigma_{pei}^{2}+\sigma_{ei}^{2}$.

Variance components were estimated using the average information restricted maximum likelihood method [11], and breeding values were estimated using the best linear unbiased prediction method [12]. The estimations were performed with the DMU package [13].

### 2.3. Validation of Genetic Evaluation

The predictive ability of the models was evaluated using a validation procedure. In the validation procedure, about 25% of the youngest pigs from the whole data were used as validation set and the rest as reference set. Two validation scenarios were considered. One scenario (valid_r1) was that the validation animals had production records but did not have NBA records, corresponding to the final selection in a pig breeding program, where the selection is made immediately after growth performance testing is completed, i.e., the candidates have growth trait records but no reproductive trait records yet. The other scenario (valid_r2) was that validation animals did not have records of both production traits and NBA, corresponding to preselection at an early stage in a pig breeding program (e.g., preselection of males at birth), where candidates have neither growth trait records nor reproductive trait records. Prediction accuracy of the models was evaluated by two measures. One is model-based accuracy (**R_m_**). R_m_ of an estimated breeding value (**EBV**) is defined as

(9) $$R_{m}=\sqrt{1-\frac{{PEV}_{i}}{A_{ii}\sigma_{a}^{2}}}$$

where PEV_i_ is the prediction error variance of EBV_i_, which is the diagonal element corresponding to EBV_i_ in the inverse coefficient matrix of the mixed model equations [14,15], and **A_ii_** is the diagonal element for individual *i* in the genetic relationship matrix.

The other is validation accuracy (**R_v_**) using the LR method [16]. The LR validation accuracy is

(10) $$R_{v}=\sqrt{\frac{Cov({\hat{a}}_{w},{\hat{a}}_{r})}{(1+\bar{F}-2\bar{f})\sigma_{a,\infty }^{2}}}$$

where ${\hat{a}}_{w}$ is the breeding value estimated using the whole data, ${\hat{a}}_{r}$ is the breeding value estimated using the reference data (also called reduced data or partial data), $\bar{F}$ is the average inbreeding coefficient of validation animals, $\bar{f}$ is the average relationship between validation animals, and $\sigma_{a,\infty }^{2}$ is additive genetic variance of validation population. Prediction bias was measured as regression of ${\hat{a}}_{w}$ on ${\hat{a}}_{r}$. A necessary condition for unbiased predictions is that the intercept does not significantly deviate from 0 (level unbiasedness) and the regression coefficient does not significantly deviate from 1 (dispersion unbiasedness).

Both R_m_ and R_v_ represent the correlation between EBV and true breeding value, a measure of selection accuracy. R_m_ is calculated based on prediction error variance (PEV) and is available for each individual EBV. But PEV is strongly depends on the model. An inappropriate model may lead to unreliable R_m_. R_v_ with the LR method was calculated based on EBV from whole data and EBV from reduced data. R_v_ also requires that the model is appropriate, but it has been shown that the R_v_ with the LR method is robust to departure from the true model [17]. However, R_v_ can measure accuracy only at the population level rather than at the individual level.

## 3. Results

Descriptive statistics are presented in Table 1. On average, Age100 was 161.90 days, LMP was 58.71%, and NBA was 13.87 piglets per litter. NBA had the largest variation and LMP had the smallest variation. The coefficients of variation were 28.05% for NBA, 9.44% for Age100, and only 1.75% for LMP. The coefficient of variation for NBA was large, probably because litter size is a composite trait that is composed of many component traits (e.g., number of ovulations, number of fertilized eggs, embryo/fetal survival, uterine capacity) and is influenced by many factors (e.g., genetics, nutrition, insemination strategy, sow management, age at first insemination, parity).

Table 2 presents variance components estimated from the single-trait models. Litter effect accounted for 10.6% of phenotypic variance for Age100, but only 4.2% for LMP. Sow permanent environment effect accounted for 7.2% of NBA phenotypic variance. The estimated heritability was 0.279 for Age100, 0.371 for LMP, and 0.076 for NBA. All variance components (litter, permanent environment, and genetic effects) in proportion to phenotypic variance were statistically significantly different from zero.

As shown in Table 3, Age100 had an unfavorable and statistically significant genetic correlation with LMP (0.308) and NBA (0.369), indicating that a pig with genetically small Age100 (fast growth) has a genetic potential to have low LMP and NBA. Genetic correlation between LMP and NBA was close to zero. Phenotypic correlations between NBA and the two growth traits were close to zero, and there was a weak but statistically significant unfavorable correlation between Age100 and LMP (0.132). The inconsistency of genetic correlation and phenotypic correlation between NBA and Age100 might be due to feed restriction after performance test based on pig body condition.

Table 4 shows accuracy and bias of genetic evaluation for NBA in validation scenario Valid_r1 where validation animals had records of Age100 and LMP but not NBA. Compared with the single-trait model, the two-trait model with NBA and Age100 led to an increase in prediction accuracy for NBA by 3.6 percentage points in R_m_ (model-based accuracy) and 5.9 percentage points in R_v_ (validation accuracy), corresponding to an increase of 8.5% and 14.9%, respectively. The two-trait model with NBA and LMP did not increase prediction accuracy for NBA. The three-trait model did not further increase prediction accuracy for NBA, compared with the two-trait model including Age100. These indicated that LMP did not provide useful information for genetic evaluation of NBA, mainly due to no genetic correlation between NBA and LMP. The level bias from the single-trait model was 0.085, which was 6.13% of the NBA mean. Multitrait models including Age100 slightly reduced the level bias for NBA. Dispersion biases were the same for the three models.

In validation scenario Valid_r2, validation animals did not have records of all the three traits. As shown in Table 5, the prediction accuracy for NBA from the two-trait model including Age100 was higher than the single-trait model by 1.8 percentage points in R_m_ and 3.8 percentage points in R_v_, corresponding to an increase of 4.2% and 9.6%, respectively. The three-trait model did not further increase prediction accuracy of NBA. LMP did not provide useful information for genetic evaluation of NBA using multitrait models. Both two-trait models with Age100 and either LMP or NBA slightly increased the prediction accuracy of Age100. Compared with the single-trait model, the three-trait model increased the prediction accuracy of Age100 by 0.9 percentage points in R_m_) and 1.8 percentage points in R_v_, corresponding to an increase of 1.8% and 3.6%, respectively. However, LMP neither benefited from a multitrait model nor contributed to genetic evaluation for NBA.

For both Valid_r1 and Valid_r2, the patterns of R_m_ in relation to different models were consistent with the pattern of R_v_, though somewhat different in absolute values. All showed an improvement of genetic evaluation for NBA by incorporating Age100 information.

Using the single-trait model, level bias was −0.659 for Age100, 0.049 for LMP, and 0.085 for NBA. In proportion to trait mean, the biases were −0.41%, 0.08%, and 6.13%, respectively. In general, multitrait models reduced level bias largely for NBA, and slightly for Age100 and LMP, except for the two-trait model with NBA and LMP. Regression coefficients ranged from 0.828 to 0.990 across traits and models. In contrast to level bias, NBA had less dispersion bias than Age100 and LMP. The differences in dispersion bias were small among single-trait and multitrait models.

## 4. Discussion

This study investigated the benefit for genetic evaluation of litter size using multitrait models including production traits. Genetic evaluations were performed for NBA, Age100, and LMP using single-trait models and multitrait models. The results showed a considerable improvement in EBV accuracy of litter size using multitrait models, compared with using the single-trait model. On the other hand, the gain from the multitrait model was relatively small for Age100, compared with the gain for NBA, and no gain for LMP. To the best of our knowledge, this is the first work that investigates the improvement of genetic evaluation for reproduction traits using information of production traits.

The benefit from a multitrait model for a particular trait depends on heritability of the trait and its correlated traits in the model as well as the genetic correlation between the trait and its correlated traits [18,19]. It has been reported that heritability of litter size is low, around 0.10 [1,2,3,4]. In this study, the estimated heritability of NBA was 0.076, lower than those in some previous studies for this trait [5,20], but slightly higher than those reported in some other studies [9,21,22]. Unlike reproduction traits, heritability of production traits is moderate or high. For growth traits such as age at slaughter weight and average daily gain, the estimated heritabilities in general range from 0.2 to 0.4 [2,8,9,20,23]. For carcass traits such as backfat thickness, loin muscle depth and area, and LMP, the estimated heritabilities in most previous reports are between 0.30 and 0.60 [2,3,8,9,24]. In the current study, heritability was 0.279 for Age100 and 0.371 for LMP, which were within the range of those reported in the literature. In line with moderate heritability for Age100 and low heritability for NBA, a two-trait model with NBA and Age100 led to a larger increase in prediction accuracy for NBA than that for Age100, compared with their respective single-trait models.

In the literature, the estimated genetic correlations between litter size and production traits are somewhat inconsistent. Most studies show low to moderate and unfavorable genetic correlations between litter size and growth, and the correlation coefficients are, in general, less than |0.40| [1,3,6,7,25]. Some studies have reported strong and unfavorable genetic correlations between litter size and growth [2,5], while one study has shown a favorable genetic correlation [20]. Genetic correlation between litter size and LMP or the related traits (backfat and loin meat) are around zero in most previous studies [1,2,3,6,25]. In the current study, the estimated genetic correlation between NBA and Age100 was 0.369, and that between NBA and LMP was 0.022. These estimates were consistent with most previous studies. There are at least four reasons that may account for the differences in estimates of genetic parameters (e.g., heritability and genetic correlation) between studies. (1) Genetic backgrounds of studied populations vary. (2) Production environments and management practices are different. (3) Statistical models and definitions may differ. (4) Estimates of genetic parameters are subject to random sampling errors. A benefit of prediction accuracy from a multitrait model with NBA and Age100 was in line with the moderate genetic correlation between NBA and Age100. In contrast, null genetic correlation between NBA and LMP explains no benefit in genetic evaluation for the two traits when using a multitrait model with the two traits. In other words, in line with genetic correlations, the genetic evaluation of NBA benefited from phenotypic information of Age100, but not from phenotypic information of LMP. The unfavorable genetic correlations between Age100 and NBA and between Age100 and LMP suggest that selection for fast growth may lead a negative correlated response for NBA and LMP, and vice versa. Therefore, a balanced breeding program is important to ensure sustainable pig production.

The benefit for a particular trait from a multitrait model depends also on the size of the phenotypic data of the trait and its correlated traits in the multitrait model. A large benefit is expected for the trait with less phenotypic information while its correlated traits have more phenotypic information. In this study, the number of records for production traits was more than double that of the NBA. As expected, NBA gained more from Age100 than Age100 gained from NBA when using a multitrait model. Furthermore, NBA gained more in scenario of Valid_r1 than Valid_r2 when using multitrait model. For Valid_r1, validation animals did not have litter size records but production trait records, corresponding to the situation of final selection at the end of performance test. For Valid_r2, validation animals had neither litter size nor production trait records, corresponding to the situation of preselection in early stage. The gain in genetic evaluation of NBA from the multitrait model in scenario Valid_r1 was much larger than that in scenario Valid_r2. The results indicate that the multitrait model is very useful for genetic evaluation of a trait for which selection animals do not have phenotypic records yet but already have phenotypic records of the correlated traits.

Multitrait models allow the use of records of correlated traits as extra information for predicting breeding values of the traits of interest and, thus, increase prediction accuracy. Therefore, multitrait models have been widely used for genetic evaluations in breeding programs, e.g., [26,27,28,29]. Many studies in dairy cattle have shown that multitrait models adding milk production traits increase accuracy and reduce bias of generic evaluation for fertility traits [30,31,32,33]. In pig, multitrait models have been used for estimating genetic parameters of reproduction and production traits. However, to date, no studies have been found in the literature on the benefits of genetic evaluation for reproductive traits in pig by adding data of production traits. To our knowledge, the current study is the first to explore the impact of multitrait models including production traits on the genetic evaluation of reproductive traits in pig. The results indicate that the accuracy of genetic evaluation of litter size can be improved by adding growth trait data using multitrait models.

Since genetic gain by selection is a linear function of genetic evaluation accuracy, a one percent increase in accuracy indicates that genetic gain by selection can be increased by one percent. For example, based on validation scenario Valid_r1 and model accuracy R_m_, the two-trait model with NBA and Age100 led to an increase in prediction accuracy for NBA by 3.6 percentage points, corresponding an increase of 8.5%, compared with the single-trait model for NBA. This indicates that selection on EBV of NBA from the two-trait model can increase genetic gain by 8.5%, compared with selection on EBV from the single-trait model. The results suggest that genetic evaluation for NBA by incorporating production trait information can efficiently increase genetic gain by selection. Based on the data in the current study, growth trait data as additional information considerably improved the accuracy of genetic evaluation of NBA. The benefit was associated with a moderate genetic correlation between NBA and Age100. The gain may be small or negligible in populations with weak genetic correlations between NBA and growth traits.

The current study also investigated prediction bias of different models. Prediction bias will not influence the ranking order of candidates when selecting on a single trait, but may change the ranking order when selecting on an index that includes many traits. The results showed very small difference in bias between single-trait model and multitrait models. However, there was a small level bias for NBA and a small dispersion bias for Age100 and LMP in all models. The reasons for the biases are not clear and need to be investigated in future study.

One of the factors that could limit the use of a multitrait model with both reproduction and production traits for routine genetic evaluation in pig breeding program is that the two group traits were in different datasets with different formats/features, e.g., an individual has a single record for a production trait while more than one record for a reproduction trait. Thus, it needs extra data editing and a specific statistical model to analyze the combined data. In previous works on estimation of genetic correlation between reproduction and production traits in pig populations, genetic correlations are estimated either for correlation between production traits and reproduction traits in a single parity, respectively [1,2,24], or ignoring environmental correlation (i.e., set to 0) between the two groups of traits [3,5,6,20]. However, genetic evaluation for reproduction traits in each single parity separately is not a good approach since it is not able to use reproduction data from all parities simultaneously and, thus, loses information. Ignoring environmental correlation between reproduction and production traits may lead to biased estimation of genetic parameters and breeding values. Oster et al. reported that (co)variances change significantly when nongenetic correlations are ignored.

The current study used a multitrait model which allowed us to account for environmental correlation of NBA with Age100 and LMP in the analysis on the data from all parities. Similar approaches have been used in some previous studies [7,34]. The results from this study and the previous studies indicate that environmental correlations between production traits (one record) and reproduction traits (multiple records) can be accounted for appropriately, and genetic evaluation of reproduction and production traits simultaneously is feasible in pig breeding programs. An alternative model was presented in [24], which uses a multitrait model where NBAs in various parities are taken as different traits.

Another limitation in using a multitrait model with both reproduction and production traits for routine genetic evaluation is an increase in data size and, thus, an increase in computational demand. However, with the rapid development of computing power, routine genetic evaluation for production and reproductive traits simultaneously using a multitrait model should no longer be a problem.

The traits in the current study were NBA, Age100, and LMP. It is necessary to estimate the genetic correlation of NBA with other traits and investigate the potential further improvement of the genetic evaluation of NBA by incorporating other traits that are genetically correlated with NBA. In addition to models used in the current study, it is worthwhile to develop and investigate better alternatives.

## 5. Conclusions

This study estimated genetic parameters of NBA, Age100, and LMP, and validated the benefit of genetic evaluation for NBA using Age100 and LMP as additional information. The results showed that NBA had a low heritability while Age100 and LMP had a moderate heritability. NBA had a moderate unfavorable correlation with Age100, and null correlation with LMP. Age100 had a moderate unfavorable correlation with LMP. These correlations suggest the importance of a balanced breeding program to ensure sustainable pig production. In consistency with the genetic correlations, the accuracy of genetic evaluation of NBA increased considerably by a multitrait model incorporating Age100 information, but there was no gain when incorporating LMP information. The multitrait model used in this study allows us to account for environmental correlation between reproduction trait and production trait where there is only one record per individual for a production trait while multiple records per individual for a reproduction trait. Therefore, the genetic evaluation of reproduction and production traits simultaneously using a multitrait model is feasible. For pig populations with genetic correlations between reproduction and production traits, a multitrait model including both reproduction and production traits is recommended for routine genetic evaluation in pig breeding programs.

## Figures and Tables

**Table 1:** Descriptive statistics for the three traits.

Trait	No.	Mean	SD	Min	Max
Age100	35,875	161.90	15.28	115.00	238.00
LMP (%)	35,875	58.71	1.03	53.24	62.36
NBA	15,636	13.87	3.89	4.00	26.00

**Table 2:** Phenotypic variance and ratios of variance components to phenotypic variance (standard errors in parentheses).

Trait	σ_p_^2^	Lit^2^	PE^2^	h^2^
Age100	196.5	0.106 (0.006)		0.279 (0.018)
LMP	0.925	0.042 (0.005)		0.371 (0.018)
NBA	12.76		0.072 (0.012)	0.076 (0.013)

**Table 3:** Correlations between the three traits (upper diagonal: genetic correlation; below diagonal: phenotypic correlation; standard errors in parentheses).

	Age100	LMP	NBA
Age100		0.308 (0.042)	0.369 (0.081)
LMP	0.132 (0.008)		0.022 (0.077)
NBA	−0.023 (0.011)	0.013 (0.011)	

**Table 4:** Accuracy, level bias, and dispersion bias of genetic evaluations for NBA in validation scenario of Valid_r1.

Parameter ^1^	Single-Trait Model	Two-Trait Model: NBA, Age100	Two-Trait Model: NBA, LMP	Three-Trait Model
R_m_	0.426	0.462	0.427	0.463
R_v_	0.395	0.454	0.397	0.451
Level bias	0.085	0.072	0.085	0.075
Disperse bias	0.986	0.983	0.989	0.987

^1^ R_m_: model-based accuracy; R_v_: validation accuracy.

**Table 5:** Accuracy, level bias, and dispersion bias of genetic evaluations for all three traits in validation scenario of Valid_r2.

Parameter ^1^	Trait	Single-Trait Model	Two-Trait Model: NBA, Age100	Two-Trait Model: NBA, LMP	Two-Trait Model: Age100, LMP	Three-Trait Model
R_m_	NBA	0.426	0.444	0.425		0.443
	Age100	0.509	0.515		0.512	0.518
	LMP	0.536		0.536	0.537	0.537
R_v_	NBA	0.395	0.433	0.393		0.429
	Age100	0.507	0.522		0.509	0.525
	LMP	0.471		0.470	0.468	0.467
Level bias	NBA	0.085	0.037	0.099		0.044
	Age100	−0.659	−0.630		−0.632	−0.609
	LMP	0.049		0.050	0.037	0.037
Disperse bias	NBA	0.986	0.976	0.990		0.966
	Age100	0.851	0.867		0.867	0.886
	LMP	0.828		0.826	0.837	0.836

^1^ R_m_: model-based accuracy; R_v_: validation accuracy.

## Data Availability

The datasets used in this study are the property of the pig industry. The data are not publicly available due to institutional restrictions. Data can be requested through the corresponding author with permission from the concerned institution.
